# Climate Change and Heat-related Illness in Older Adults: Implications for Emergency Medicine

**DOI:** 10.5811/westjem.54008

**Published:** 2026-08-17

**Authors:** Erin L. Simon, Kevin Watkins, Stephen Meldon

**Affiliations:** * Cleveland Clinic Akron General, Department of Emergency Medicine, Akron, Ohio; † Cleveland Clinic, Department of Emergency Medicine, Cleveland, Ohio

## Abstract

Climate change is increasing the frequency, duration, and intensity of extreme heat events, creating a growing public health threat for older adults. Age-related physiologic decline, comorbid chronic diseases, polypharmacy, and social vulnerability all contribute to reduced heat tolerance and higher rates of heat-related morbidity and mortality. Emergency departments (ED) are increasingly encountering older adults presenting with minor heat illness, heat stroke, and exacerbations of chronic illnesses triggered by heat exposure. This review synthesizes current evidence describing why older adults are disproportionately affected by extreme heat, highlights key social and environmental determinants of vulnerability, and outlines clinical and public health strategies that emergency clinicians and EDs can assist with to reduce risk. As global temperatures rise, proactive prevention, early recognition, and coordinated community interventions are essential to protect this high-risk population.

## INTRODUCTION

Climate change has led to a rise of 1.5 °C in mean global temperature since the Industrial Revolution, with projected increases of 2.5–2.9 °C by the end of this century^1^ and is accelerating the burden of heat-related illness worldwide, with older adults among the most affected. Rising average temperatures and more frequent, severe, and prolonged heat waves have led to marked increases in heat-related morbidity and mortality. The majority of deaths due to heat waves globally are in those > 65 years of age.^2^ In adults ≥ 65 years, heat-related deaths have risen by more than 50% in the past two decades, with climate change accounting for a substantial share of warm-season mortality.^3^,^4^ In the United States, those > 65 years of age account for 40% of all heat-related deaths.^5^ In addition, older individuals are likely to constitute a higher percentage of the population, with those at least 69 years old projected to increase from 11% in 2024 to 17% in 2050. This population is particularly concentrated in the Northeast, Midwest, and South Atlantic.

Shared socioeconomic pathways are climate-population scenarios used to project how demographic change, economic development, and inequality may influence future heat exposure and health risk.^6^ In this context, these models suggest that older adults, particularly those with medical comorbidity, limited resources, or reduced access to cooling, will face progressively greater heat exposure and heat-related health consequences over time.^4^,^7^–^10^ For emergency clinicians, the relevance of these models is that they anticipate a growing number of older adults at risk for heat-related illness and heat-triggered exacerbations of chronic disease, underscoring the need for improved recognition, prevention, and systems-level preparedness.^11^

Older adults face unique vulnerabilities due to impaired thermoregulation, higher rates of chronic disease, polypharmacy, and use of medications that impair heat dissipation, reduced physiologic reserve, and social factors such as isolation mobility issues that limit access to cooling resources.^12^,^13^ The emergency department (ED) is often the first point of contact for patients experiencing heat-related illness or exacerbations of heat-sensitive conditions. This review summarizes the physiologic, pharmacological, environmental, and social contributors to heat vulnerability in older adults and provides guidance for emergency clinicians caring for this growing at-risk population.

## PHYSIOLOGIC AND PHARMACOLOGIC VULNERABILITY OF OLDER ADULTS

Aging significantly reduces the body’s ability to thermoregulate. Older adults experience diminished sweat production, reduced skin blood flow, impaired cardiovascular responses, and decreased renal concentrating ability, all of which contribute to heat retention.^14^,^15^ Additionally, older adults underestimate their vulnerability to extreme heat.^15^

Comorbidities common in older age, such as cardiovascular disease, diabetes, chronic kidney disease, chronic respiratory illness, obesity, and dementia, further limit thermoregulatory efficiency and increase the risk of dehydration, hypotension, and organ dysfunction during heat exposure.^13^,^16^ Heat exposure not only causes classic heat-related illnesses but also exacerbates underlying chronic conditions, such as diabetes, hypertension, chronic kidney disease, and cardiovascular disease, increasing the risk of hospitalization and death. Studies consistently report an increase in cardiovascular and respiratory mortality in elderly persons during hot days.^17^

Polypharmacy is another major driver of vulnerability. Frequently prescribed medications, including diuretics, beta-blockers, calcium channel blockers, anticholinergics, antipsychotics, antihistamines, and antidepressants, can impair sweating, alter fluid balance, blunt cardiovascular compensation, or affect cognitive awareness, thereby increasing susceptibility to heat-related illness.^8^,^18^–^21^

Taken together, age-related physiologic decline, chronic medical conditions, and medication effects substantially elevate the risk of heat exhaustion, heat stroke, and decompensation of existing illnesses during extreme heat events. Key physiologic changes and medication classes that increase heat vulnerability in older adults are summarized in Table 1.

## ENVIRONMENTAL AND SOCIAL DETERMINANTS OF HEAT VULNERABILITY

Heat risk in older adults is strongly shaped by environmental, demographic, and socioeconomic factors. Individuals living in poverty or on fixed income, in older housing without air conditioning, or neighborhoods with dense infrastructure and minimal green space (known as urban heat islands) face disproportionate exposure to extreme heat.^5^,^19^ Additionally, older Americans living in northern cities may be particularly vulnerable to the effects of heat waves due to the relative lack of acclimatization and air conditioning.^22^ Geographic “hotspots” where older Americans may be sensitive are the deep South and upper Midwest.^11^

Social isolation, limited mobility, and lack of access to transportation or community cooling centers further increase vulnerability; in fact, isolation has been identified as a key risk factor for death during extreme heat events.^23^ Those living in isolation, especially with cognitive impairments, may not receive or understand the severity of emergency information. Marginalized racial and ethnic groups face additional risk due to structural inequities, including limited access to healthcare, fewer cooling resources, and residence in hotter urban microclimates.^8^

These intersecting vulnerabilities amplify the likelihood of heat-related hospitalizations and deaths in older populations. Recognizing these social and environmental risks is essential for effective prevention and targeted outreach during heat waves.

Population Health Research Capsule

What do we already know about this issue?

*Older adults account for most heat-related deaths due to impaired thermoregulation, chronic disease, medications, and social vulnerability*.

What was the research question?

*Why are older adults especially vulnerable to extreme heat, and what can EDs do to reduce risk?*

What was the major finding of the study?

*Heat-related deaths in adults ≥65 years increased >50% over two decades*.

How does this improve population health?

*Identifies prevention, early recognition, and community interventions to reduce heat-related illness and death in older adults*.

## CLINICAL IMPLICATIONS FOR EMERGENCY MEDICINE

Clinical implications of extreme heat for emergency medicine include both system- and department-level, and patient-level considerations. Associations between extreme heat events and increases in ED visits and hospitalization are well documented. One study noted an increase of nearly 8% in the relative risk for ED visits for any cause, equating to 24 excess visits per 100,000 persons at risk per day.^24^ An analysis of approximately 50 million summertime admissions showed an increased rate of admission correlating to increases in daily maximum heat index across the U.S.^25^ Mortality also increases with significant heat events, with high ambient temperatures and multiple heat waves causing an estimated 61,000 heat-related deaths in Europe in 2022.^26^

Clinically, older adults often present with atypical or subtle manifestations of heat-related illness. Confusion, weakness, decreased oral intake, falls, syncope, or worsening of chronic conditions may precede classic symptoms such as hyperthermia or anhidrosis; additionally, classically described features, such as anhidrosis, are not always present. The symptoms often overlap with or occur simultaneously with other common conditions, such as sepsis. During heat waves, emergency physicians should maintain a high index of suspicion, particularly in patients with known comorbidities or those taking heat-sensitive medications that increase the risk of heat illness.

Rapid assessment, including accurate core temperature measurement and prompt management such as active cooling, volume resuscitation, electrolyte correction, and careful review of medications, are critical to preventing progression to heat stroke and multiorgan dysfunction. Acute mild and moderate heat-related illnesses include heat rash (pruritic papules resulting from blocked eccrine sweat glands), heat cramps (painful involuntary muscle cramping resulting from sweating-induced dehydration and electrolyte imbalances), heat edema (which may occur in the hands and feet), heat syncope (generally associated with prolonged standing or a change to a standing position in the setting of hot temperatures), and heat exhaustion. Heat exhaustion typically presents as weakness, dizziness, nausea, headache, and muscle cramps, with heavy sweating and rapid pulse evident; the core body temperature may be elevated, but mental status remains normal. Heat stroke is a severe form of heat illness, in contrast to heat exhaustion, involves alterations in central nervous system functioning in association with a core body temperature exceeding 40 °C. Heat stroke is a true medical emergency and can rapidly progress to multiorgan failure and death. Recent clinical reviews provide an excellent summation of the full spectrum of heat-related illnesses, associated medications effects, and evidence-based treatment approaches.^3^,^18^ The clinical presentation and treatment of heat illness, from mild heat edema and heat syncope to heat exhaustion and the more severe heat stroke, should be familiar to all emergency clinicians.

Emergency clinicians also play a role in prevention by educating patients and caregivers, identifying those at risk for recurrent heat-related illness, and coordinating with outpatient clinicians and community resources. Emergency physicians can specifically provide information on cooling centers and discuss the risks and benefits of certain medications during heat waves. Common and atypical presentations of heat-related illness in older adults, along with a high-level emergency department approach, are summarized in Table 2.

## PREVENTION AND PUBLIC HEALTH STRATEGIES

Heat-related illnesses in older adults are highly preventable. Effective strategies include anticipatory guidance before heat waves, early warning systems, accessible cooling centers, home air-conditioning assistance, and social outreach programs that check on isolated older adults during periods of extreme heat.^16^,^19^,^20^

Clinicians can help reduce risk by reviewing medications during hot weather, encouraging adequate hydration, promoting awareness of early heat illness symptoms, and advising patients to avoid outdoor or strenuous activities during peak heat hours. In addition, media interviews with emergency clinicians are a frequent request during heat spells and can be effective at reaching older audiences over a large local area. Furthermore, emergency physicians can provide knowledge of local resources, such as location of cooling centers, during heat waves.

Partnerships between EDs, public health agencies, emergency medical services, and community organizations are essential to building climate resilience and protecting vulnerable older adults.

## CONCLUSION

Older adults face disproportionate and growing risks from climate change-related heat exposure. Physiologic decline, chronic medical illness, medication effects, and social vulnerability all contribute to increased susceptibility to heat-related morbidity and mortality. Emergency clinicians are uniquely positioned to recognize at-risk individuals, initiate timely management, and support prevention efforts. As climate change accelerates, integrating heat-risk awareness into emergency care and collaborating with public health systems will be crucial to safeguarding the health of the growing demographic of older adults.

## Figures and Tables

**Table 1:** Physiologic and pharmacologic factors contributing to heat vulnerability in older adults.

Category	Factor	Mechanism	Emergency medicine relevance
Physiologic factors	Diminished sweat production	Reduced evaporative cooling	Higher risk of heat retention and delayed heat dissipation
	Reduced skin blood flow	Impaired peripheral vasodilation limits heat transfer to skin	Less effective thermoregulation during heat exposure
	Impaired cardiovascular response	Reduced cardiac reserve limits circulatory compensation	Greater risk of hypotension, syncope, and decompensation
	Decreased renal concentrating ability	Reduced ability to conserve water and maintain fluid balance	Increased risk of dehydration, electrolyte abnormalities, and acute kidney injury
	Reduced physiologic reserve	Lower tolerance for thermal stress	More rapid deterioration with modest heat exposure
	Chronic comorbid disease	Cardiovascular, metabolic, renal, pulmonary, and cognitive disease may impair adaptation to heat	Higher risk of atypical presentation, delayed recognition, and worse outcomes
Pharmacologic factors	Diuretics	Increase fluid loss and electrolyte derangements	Higher risk of dehydration, hypotension, and acute kidney injury
	Beta-blockers	Blunt cardiovascular compensation	Reduced physiologic response to heat stress
	Calcium channel blockers	May impair hemodynamic adaptation	Possible worsening of heat-related instability
	Anticholinergics	Decrease sweating	Impaired heat dissipation and greater overheating risk
	Antipsychotics	Disrupt thermoregulation and cognition	Delayed recognition and more severe presentation
	Antihistamines	May impair sweating and cause sedation	Reduced symptom awareness and heat tolerance
	Antidepressants	May alter sweating, cognition, or fluid balance	May contribute to subtle or mixed presentations

**Table 2:** Heat-related illness in older adults: common presentations and emergency department approach.

Category	Typical features in older adults	Emergency department approach
Atypical early presentation	Confusion, weakness, decreased oral intake, falls, syncope, or worsening of chronic disease; classic findings such as anhidrosis may be absent	Maintain high suspicion during heat events, especially in patients with comorbidities or heat-sensitive medications; obtain accurate core temperature and assess volume status, electrolytes, and end-organ injury
Heat rash / heat edema / heat cramps	Pruritic rash, dependent swelling, painful muscle cramping	Supportive care, cooling, oral or IV hydration as needed, and correction of electrolyte abnormalities
Heat syncope	Brief loss of consciousness or presyncope, often after prolonged standing or abrupt standing in hot conditions	Supine positioning, cooling, hydration, and evaluation for alternative or concurrent causes of syncope
Heat exhaustion	Weakness, dizziness, nausea, headache, muscle cramps, heavy sweating, tachycardia; temperature may be elevated, but mental status remains normal	Remove from heat, active cooling, fluid resuscitation, electrolyte correction, medication review, and reassessment for progression
Heat stroke	Core temperature typically > 40 °C with altered mental status or other CNS dysfunction; may rapidly progress to multiorgan failure	Immediate emergency treatment with rapid cooling, aggressive supportive care, resuscitation, and close monitoring for organ dysfunction
Exacerbation of chronic illness	Decompensation of cardiovascular, renal, respiratory, or metabolic disease during heat exposure	Evaluate for concurrent heat-related illness while treating the underlying exacerbation; review contributing medications and disposition carefully
Disposition and prevention	Older adults may remain vulnerable after apparent stabilization	Provide counseling on hydration, medication risks, early symptoms, and avoidance of peak heat; connect patients and caregivers with cooling centers and community resources

*CNS*, central nervous system; *IV*, intravenous.
